# Elimination of Ligation Dependent Artifacts in T4 RNA Ligase to Achieve High Efficiency and Low Bias MicroRNA Capture

**DOI:** 10.1371/journal.pone.0094619

**Published:** 2014-04-10

**Authors:** Yunke Song, Kelvin J. Liu, Tza-Huei Wang

**Affiliations:** 1 Biomedical Engineering Department, Johns Hopkins University, Baltimore, Maryland, United States of America; 2 Mechanical Engineering Department, Johns Hopkins University, Baltimore, Maryland, United States of America; 3 Circulomics Inc, Baltimore, Maryland, United States of America; Naval Research Laboratory, United States of America

## Abstract

Adapter ligation is a critical first step in many microRNA analysis methods including microarray, qPCR, and sequencing. Previous studies have shown that ligation bias can have dramatic effects on both the fidelity of expression profiles and reproducibility across samples. We have developed a method for high efficiency and low bias microRNA capture by 3′ adapter ligation using T4 RNA ligase that does not require pooled adapters. Using a panel of 20 microRNA, we investigated the effects of ligase type, PEG concentration, ligase amount, adapter concentration, incubation time, incubation temperature, and adapter design on capture efficiency and bias. Of these factors, high PEG% was found to be critical in suppressing ligation bias. We obtained high average capture efficiency and low CV across the 20 microRNA panel, both in idealized buffer conditions (86%±10%) and total RNA spiking conditions (64%±17%). We demonstrate that this method is reliable across microRNA species that previous studies have had difficulty capturing and that our adapter design performs significantly better than the common adapter designs. Further, we demonstrate that the optimization methodology must be specifically designed for minimizing bias in order to obtain the ideal reaction parameters.

## Introduction

MicroRNA and other small RNA have added a new dimension to the connection between genotype and phenotype. These new mechanisms for gene expression regulation have led to a wealth of studies detailing the pervasive roles of microRNA in areas such as developmental biology [1], [2], [3], stem cell biology [4], [5], [6], cancer [7], [8], [9], [10], and plant genomics [11], [12], [13]. MicroRNA are studied both to elucidate their roles in fundamental mechanistic pathways as well as to develop novel disease biomarkers [14], [15] and therapeutics [16], [17]. The majority of microRNA assay techniques been adapted from existing mRNA analysis methods. However, due to their short length, the first step of nearly all microRNA assays is to modify the microRNA through reverse-transcription [18], [19], [20], poly(A)-tailing [21], [22] or ligation [23], [24]. Among these methods, microRNA capture through adapter ligation is a pervasive first step in many PCR- [15], [25], [26], microarray- [27], [28], [29], bead-[20] and sequencing- based assays [23], [30], [31].

Due to the rising popularity of 2nd generation sequencing for small RNA detection and discovery, a number of studies have sought to benchmark microRNA expression profiles across various detection platforms and systematically look for sources of bias [23], [32], [33], [34], [35]. Sequencing based methods are enjoying rising popularity due to their ability to identify small RNA species *de novo* and due to their ability to distinguish closely related isoforms. Although these sequencing approaches typically involve many sequential enzymatic steps including reverse transcription, PCR amplification, ligation, and poly(A) extension, a number of recent studies have pinpointed adapter ligation as the main contributor to expression profile bias [36], [37], [38], [39].

Ligation bias is critical because it underlies such a large number of microRNA analysis methods. Ligation can introduce two distinct levels of bias to microRNA expression profiles. First, bias can be introduced across samples when different adapters are used on different individual samples. Alon *et al* showed that consistent differential expression profiles can be seen across samples when the same adapter sequence is used but that large variations are seen when different adapter sequences are used even within the same sample [37]. This can be a significant problem when comparisons are made across assay platforms that use different adapter sequences or when adapters are used to barcode individual samples such as in multiplexed deep sequencing applications. Second, bias can be introduced within each sample across various microRNA species, distorting the resultant expression profiles. Hafner *et al* demonstrated that microRNA species can appear over or under-expressed by multiple orders of magnitude due to biases in ligation efficiency [39], [40]. This is less of an issue in differential expression analysis but is a significant issue when comparisons are made across microRNA species to rank expression levels. Though the majority of recent studies have examined bias in the context of sequencing based methods, this ligation bias will have similar effects on other miRNA assays such as PCR and array based methods that incorporate 3′ ligation.

Recent studies have sought to identify the cause of ligation bias and remediate it [36], [37], [38], [40], [41], [42]. All of these recent studies have focused on improving adapter design to reduce bias. Jayaprakash *et al* found that two terminal bases on the 3′ adapter can have dramatic effect on ligation efficiency [36]. Zhuang *et al* and Hafner *et al* demonstrated that secondary structure interactions can contribute significantly to variations in ligation efficiency [40], [41]. Remediation strategies have included optimization of ligase choice, optimizing secondary structure interactions, and incorporating adapter pools [36], [38], [41], [42], [43]. To our knowledge, few studies other than Zhang *et al* have investigated the optimization of reaction conditions for bias suppression [43]. Furthermore, nearly all have resorted to the use of randomized adapter pools [36], [38], [41], [42], [43]. This may be due to the commonly held perception that T4 RNA ligase is inherently biased and difficult to use.

In this study, we have designed a microRNA capture method based on 3′ adapter ligation that achieves very high efficiency (86% AVG) and low bias (10% SD) across all microRNA species tested. High efficiency capture is demonstrated even with microRNA that previous studies have had difficulty capturing and even based on the standard 3′ modban adapter that previous studies have shown to exhibit high ligation bias. Using a panel of 20 microRNA, we studied key assay parameters such as PEG%, enzyme selection, adapter saturation, and design and show that they can be used to suppress bias and nearly eliminate ligation preference given suitable optimization methodology. We demonstrate that optimization must be done in the presence of total RNA using a microRNA panel to minimize global bias, as erroneous conditions can be found if optimization is done using only a single microRNA or in synthetic conditions.

## Material and Methods

### Adapter Oligonucleotides

MicroRNA targets were synthesized by Integrated DNA Technologies (Coralville, IA). They consist of HPLC purified, DNA oligonucleotides with 5′-Ph and either 3′-ddC blocking group or 3′-Cy5 label. The adapters are based on modified 3′ modban adapters [44]. The adapters were enzymatically pre-adenylated with T4 RNA ligase using a process similar to Thomson *et al* [28]. Additional adapters were also synthesized for comparison purposes based on the SR1 and SR1-S sequences reported by [41].

### Synthetic microRNA

MicroRNA were synthesized by Integrated DNA Technologies (Coralville, IA). The sequences were taken from miRBase (www.mirbase.org) and are listed in **Table S1 in File S1**. They targets consist of HPLC purified RNA oligonucleotides derivatized with 3′-OH and 5′-Cy3 end groups.

### Ligation Protocol

Unless otherwise indicated, ligation was performed by mixing 1.25 µL of 2 µM adenylated adapter, 1 µL of T4 RNA Ligase buffer (New England Biolabs, Ipswich, MA), 5 µL of 50% PEG8000, 1 µL of synthetic target, 0.5 µL of total RNA, 1 µL of T4 RNA Ligase 2 truncated K227Q (New England Biolabs, Ipswich, MA) and water into a 20 µL reaction volume. The reaction was then incubated at 25°C for 4 hours and heat denatured at 65°C for 20 minutes in a thermal cycler. In the experiments where different ligases were investigated, T4 RNA Ligase 2 truncated, T4 RNA Ligase 2 truncated R55K K227Q, and Thermostable 5′ App DNA/RNA Ligase were all obtained from New England Biolabs. In spiking experiments, 500 ng of human brain total RNA (Ambion, Austin, TX) was added to each sample.

### PAGE Analysis

The samples were analyzed on precast 15% TBE-urea polyacrylamide gels (Bio-Rad, Hercules, CA). 5 µL of sample was mixed with 5 µL of loading buffer and heated for 5 minutes at 95°C. The sample was then loaded into the gel and run for either 30 min or 50 min at 300V. The separated gels were scanned using a Typhoon 9410 variable mode imager (GE Healthcare, Piscataway, NJ). The gel images were analyzed using ImageQuant (GE Healthcare, Piscataway, NJ) to obtain lane profiles. These profiles were then curve-fit with Gaussian curves using Origin (OriginLab, Northampton, MA) to precisely determine band position and intensity.

## Results and Discussion

### Bias in Ligation Based microRNA Capture

MicroRNA consist of short RNA sequences that are typically 17–23 nt in length. Due to their short length, 5′ and/or 3′ adapter ligation is often used to label, capture, or lengthen the microRNA before downstream detection. A number of studies have suggested a pooled adapter approach to average out the intrinsic effects of ligation bias. However, by improving ligation reaction design and optimization methodology, we have developed a ligation based method that achieves high efficiency and low bias microRNA capture without the need for adapter pools.

**Figure 1:**
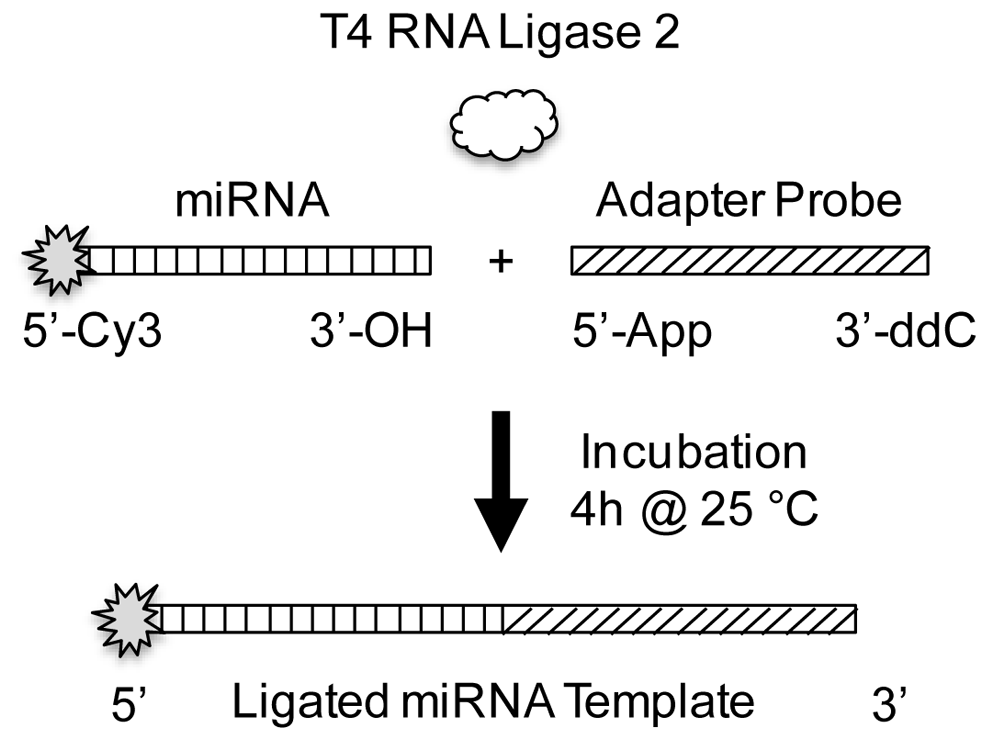
Schematic illustration of microRNA capture by 3′ adapter ligation. The 19 nt, enzymatically pre-adenlyated adapter is ligated to the 3′ OH of microRNA using T4 RNA ligase 2. The reaction is run at 25°C for 4 hours in the absence of ATP. In order to characterize capture efficiency, the microRNA is end labeled with Cy3. The 3′ end of the adapter is blocked by –ddC, a fluorophore, or other moiety to prevent the formation of concatemers and circularized products.

As shown in **Figure 1**, an adapter oligonucleotide is ligated to the 3′-OH of each microRNA using T4 RNA ligase 2. To reduce side product formation, the adapter is first enzymatically pre-adenylated such that the ligation reaction can be performed in the absence of ATP. This prevents the microRNA in the sample from undergoing self-circularization, self-polymerization, and ligation to RNA species other than the adapter. Second, the 3′ end of the adapter is blocked with dideoxycytosine (ddC), a fluorophore, or other moiety to prevent self-circularization and adapter concatenation. Finally, a recombinant mutant ligase is used. These enzymes lack the domain necessary for ATP incorporation and contain point mutations that further suppress side product formation. Such an approach is commonly used in miRNA analysis ahead of reverse transcription [15], [24], [44], [45], [46]. Using this general reaction design, we investigated the effects of specific reaction conditions in suppressing ligation bias.

In order to characterize overall ligation efficiency and ligation bias, we synthesized a panel of 20 representative microRNA. Ten of the microRNA were selected based on their reported roles as important cancer-related microRNA (let-7a, miR-16, miR-21, miR-26a, miR-29b, miR-34a, miR-15a, miR-17p, miR-92a, and miR-155) [9], [10], [47]. Seven of the microRNA were chosen to enable comparison against recent publications (miR-31, miR-338, miR-567, miR-4803, miR-5183, miR-712, and miR-106b). For example, Zhuang *et al* reported difficulties capturing miR-4803, miR-5183, and miR-567 while Hafner *et al* reported low capture efficiencies for miR-31, miR-712, and miR-338 [40], [41]. Jayaprakash *et al* reported that miR-106b could not be captured consistently under any of their experimental conditions. The final three microRNA were randomly selected (miR-25, miR-125b, miR-19b). Each target microRNA was labeled with a Cy3 dye at the 5′ end to enable quantification of ligation efficiency under spiking conditions in total RNA. The reaction products were analyzed using denaturing PAGE, and the gels were scanned using a multimode imager. Image analysis was then used to obtain band positions and DNA quantity. Using this PAGE analysis method, we obtain excellent quantification and reproducibility. Quantification is linear from <5 amols to >10 pmols with an experiment to experiment CV of 10% (**Figure S1 in File S1**).

### Ligase Type

**Figure 2:**
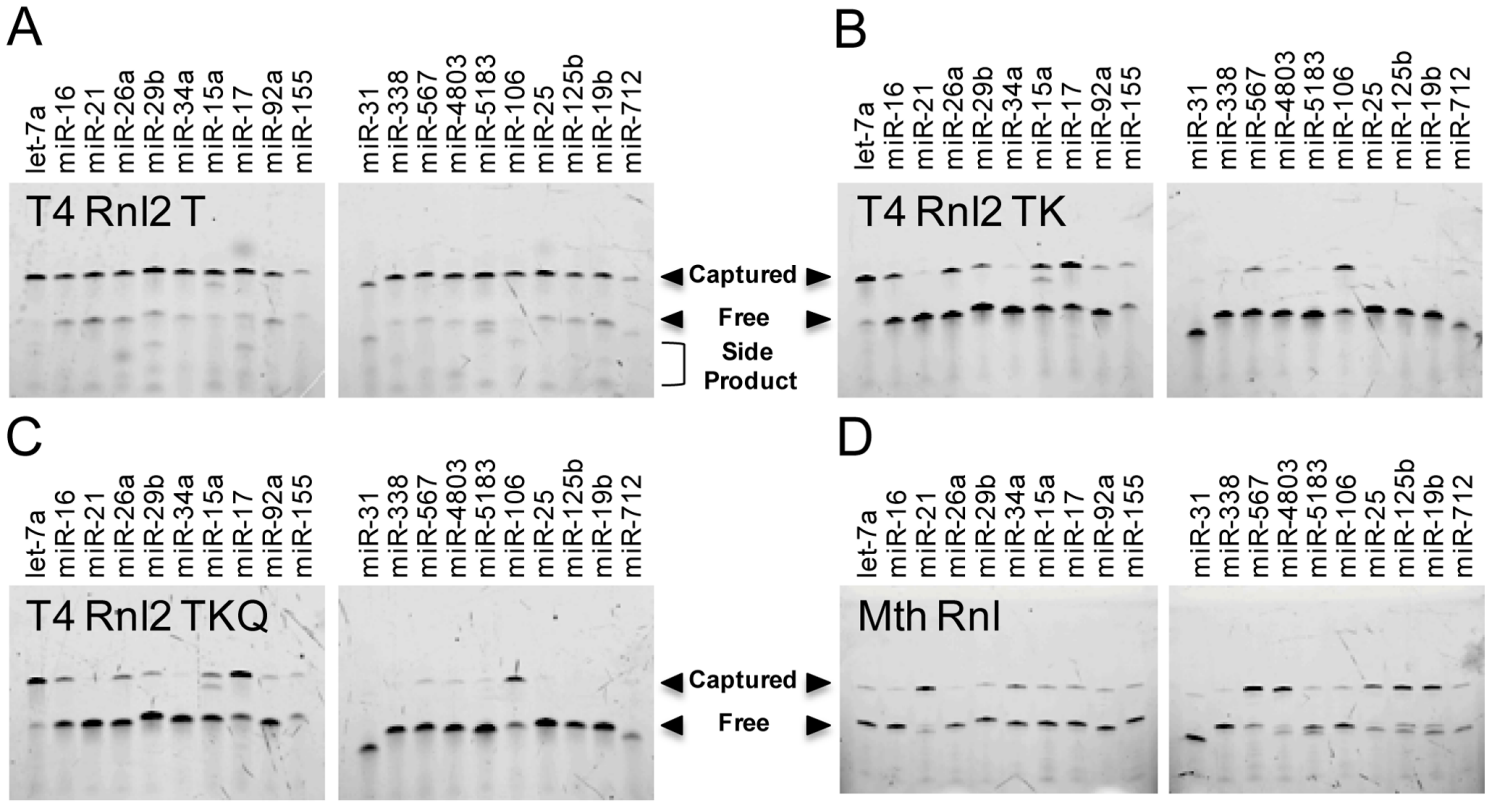
MicroRNA capture was performed with 4 different ligases using the vendor recommended protocols to compare capture efficiency across 20 different microRNA. The ligation products were analyzed by 15% denaturing urea-PAGE. Capture efficiency was determined by performing a Cy3 scan and comparing the intensities of the ∼40 nt captured microRNA band versus the ∼20 nt free microRNA band. T4 RNA Ligase 2 truncated (T4 Rnl2 T) had high average capture efficiency and low bias but many randomly sized background products. The point mutant enzymes T4 RNA Ligase 2 truncated K227Q (T4 Rnl2 TK) and T4 RNA Ligase 2 truncated KQ (T4 Rnl2 TKQ) had decreased side product formation but also lower average capture efficiency and higher bias. Thermostable 5′ App DNA/RNA Ligase (Mth Rnl), which was performed at 65°C instead of 25°C, had similar average capture efficiency and bias but with distinct ligation efficiency pattern.

The first parameter we investigated was ligase type, as the ligases themselves likely have different intrinsic bias. **Figure 2** shows a denaturing PAGE analysis of adapter ligation on our 20 microRNA test panel using 4 different RNA ligases. For each ligase, we used the manufacturer recommended ligation conditions. As each microRNA is labeled with Cy3, the capture efficiency was easily quantified using image analysis to compare the band intensities between the free microRNA band at ∼20 nt and the ligated microRNA band at ∼40 nt. A quantitative analysis of ligation efficiency for each microRNA and each ligase is provided in **Figure S2 in File S1**.

T4 RNA ligase 2 truncated (T4 Rnl2 T) is a mutant enzyme that lacks the domain necessary for ATP incorporation, which should significantly reduce side product formation when used with pre-adenylated adapters in the absence of ATP [48]. As evidenced by the bright uniform bands in Figure 2A, this enzyme gave high ligation efficiency (66% AVG) and low bias (11% SD) with every microRNA species being captured at >40%. However, a significant number of background products can also be seen on the gel. A number of unique and randomly sized bands, which run faster than both the captured microRNA and the free adapter, are visible in each lane. It is unclear what these products are as typically only circularized products can run faster than linear products of the same size, but the same 5′-Cy3 that enables visualization should also prevent the formation of such circularized products.

T4 RNA ligase 2 truncated K227Q (T4 Rnl 2 TK) contains a point mutation that is designed to further reduce side product formation [48], [49]. This effect is clearly seen in Figure 2B, where side product formation is suppressed and only the desired products are visible. Smaller, randomly sized bands are no longer apparent. Yet, the overall ligation efficiency has decreased dramatically (20% AVG) and ligation bias across the microRNA panel is quite significant (25% SD). Six microRNA were captured at <2% efficiency.T4 RNA ligase 2 truncated KQ (T4 Rnl 2 TKQ) is a double-point mutant that is also designed to have low side product formation but with increased ligation activity that is restored to the levels of T4 Rnl2 T [48]. In our experiments, little difference was seen between T4 Rnl 2 TK and T4 Rnl 2 TKQ, which had a capture efficiency of 17%±24%. T4 Rnl2 TKQ had 7 microRNA that were poorly captured at <2% efficiency.

Finally, we tried Thermostable 5′ App DNA/RNA Ligase (MthRnl) from New England Biolabs, which is a point mutant of RNA ligase isolated from *Methanobacterium thermoautotrophicum*. This thermostable ligase is unable to incorporate ATP and works optimally at 65°C. Ligation at an elevated incubation temperature could serve to reduce bias from secondary structure interactions. Though the same adapters and microRNAs were used, a different pattern of ligation bias was seen. This likely arose from ligation preferences intrinsic to the ligase itself. In addition, despite the higher reaction temperature, neither ligation efficiency (30% AVG) nor bias (28% SD) was significantly improved over the T4 Rnl2 variants.

Although T4 Rnl2 T had high ligation efficiency and low bias, the large amounts of background products complicate downstream processes and were deemed unacceptable. We chose to use T4 Rnl2 TK moving forward due to the belief that it would be easier to increase reaction efficiency and reduce bias than to suppress side product formation.

### PEG Levels

Additives such as polyethylene glycol (PEG) [50], [51] and dimethyl sulfoxide (DMSO) [24], [27], [28] are commonly added to ligation reactions to increase reaction efficiency. In our preliminary experiments, we saw minimal effect with DMSO addition (data not shown). However, we saw dramatic effects on ligation efficiency and bias due to PEG. PEG is thought to increase molecular crowding [50], [51], [52], and many studies as well as manufacturer protocols have recommended ∼15% PEG as an ideal concentration [15], [24]. Ligation efficiency is said to plateau or even decrease at high PEG levels.

**Figure 3:**
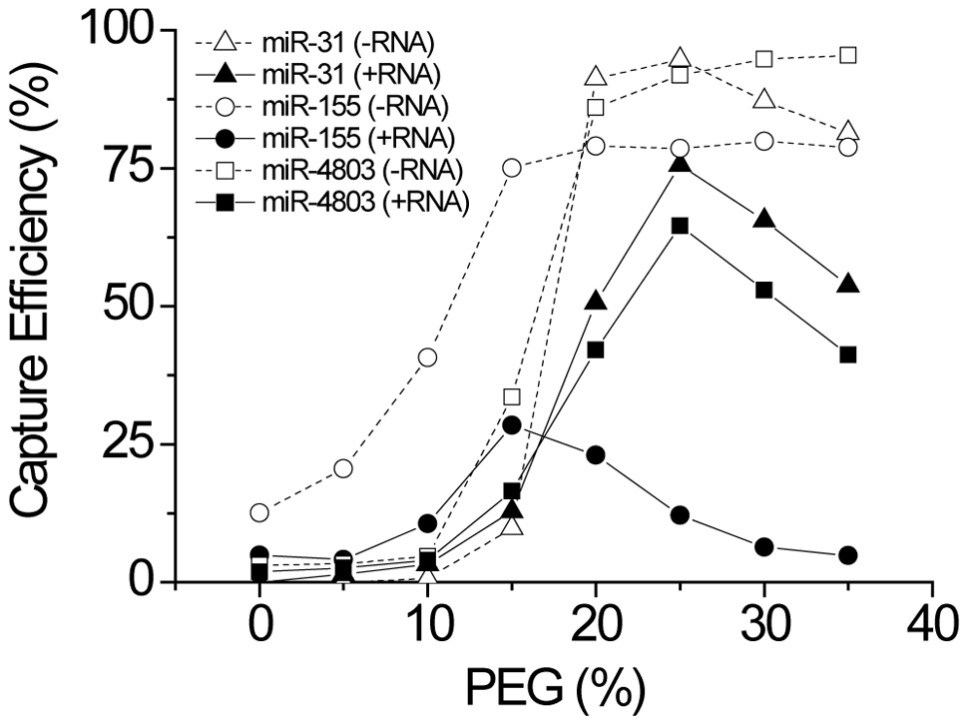
Comparison of ligation efficiency as a function of PEG percentage for miR-31, miR-155, and miR-4803 in idealized buffer (open markers) and total RNA spiking conditions (filled markers). miR-155 and miR-4803 display similar behavior with respect to PEG percentage while miR-31 behaves distinctly. Different behavior is also seen between idealized buffer conditions and total RNA spiking conditions, illustrating the importance of optimization methodology in extrapolating assay performance. Optimizations performed using a single microRNA species in idealized buffer may not extrapolate to other microRNA under actual assay conditions.

Based on the initial results of Figure 2B, we optimized the effects of PEG on a subset of our 20 microRNA panel. We tested three microRNAs that were initially poorly captured by T4 Rnl2 TK, miR-31 (2.5% capture efficiency), miR-155 (28% capture efficiency), and miR-4803 (3.5% capture efficiency). 10 nM of each microRNA was individually spiked into 500 ng of total RNA. This represents about ∼3–4 million microRNA copies per cell and is sufficient to approximate the aggregate expression of all microRNA within the cell. As the PEG concentration was varied from 0–35%, **Figure 3** and **Figure S3 in File S1** show that the capture efficiency generally increased as a function of PEG level and then decreased at high PEG levels. MiR-31 and miR-4803 behaved similarly, reaching the highest capture efficiency at 25% PEG, while miR-155 reached maximum efficiency at 15% PEG. The variation seen between these microRNA underscores the importance of optimizing reaction conditions across multiple microRNA species. The ideal conditions for a single microRNA do not necessarily extrapolate to other microRNA.

When the microRNAs were spiked into idealized buffer conditions rather than total RNA, a different behavior was seen. Maximum capture efficiency was reached at lower PEG levels with miR-31 and miR-4803 behaving similarly again, reaching plateau at 20% PEG, and miR-155 reaching plateau at 15% PEG. The overall ligation efficiencies also increased significantly, particularly for miR-155. Total RNA likely contains inhibitors that prevent ligation from reaching completion such as: 1) RNA species that bind and sequester the microRNA, 2) RNA species that ligate competitively to the microRNA, 3) RNA species that ligate competitively to the adapter, 4) RNA species that ligate competitively to each other, and 5) inhibitors of the ligase. Though our and reaction design should minimize effects 2), 3), and 4), the large amount of background RNA can still occupy the ligase binding site even if the actual ligation cannot occur (i.e. ligase shaking hands but not making deals). In addition, the decrease in ligation efficiency seen at high PEG levels under spiking conditions is also absent or greatly reduced under idealized buffer conditions. It is unclear what the exact mechanism of PEG is and why different effects would be seen with and without total RNA spiking. Furthermore, the discrepancy in ligation behavior across the three microRNA also appears to decrease, with all three microRNA being well captured at 20% PEG.

This data illustrates that the optimal assay parameters determined using 1) a single microRNA vs. a panel and 2) in idealized buffer vs. spiking conditions are quite different, highlighting the critical importance of optimization methodology and design. Most previous publications, as well as manufacturer protocols, have recommended 12–15% PEG. At this PEG level, only miR-155 is optimally captured under spiking conditions. In spiking conditions, ligation at 20–25% PEG is optimal whereas ligation at the recommended 15% PEG leads to very low efficiency. Of all the parameters investigated, PEG had the most dramatic effect on ligation bias and efficiency.

### Adapter Concentration and Ligase Amount

Next we tested the effects of adapter concentration and ligase amount in conjunction with one another. Adapter concentration must be in excess to the ligated species to drive ligation forward. In addition to microRNA, samples often contain other RNA species such as mRNA, rRNA, and siRNA that can also be ligated. Having too few adapters will limit the ligation efficiency and increase bias. Having too large an excess of adapters will promote side product formation. In addition, large excesses of free adapters may also complicate downstream assay processes. The ligase amount also needs to be sufficient to obtain a high ligation efficiency in a reasonable amount of time. However, high ligase amounts can promote side product formation as well. Practically speaking, ligase is the most expensive reaction component and should be minimized to reduce costs.

**Figure 4:**
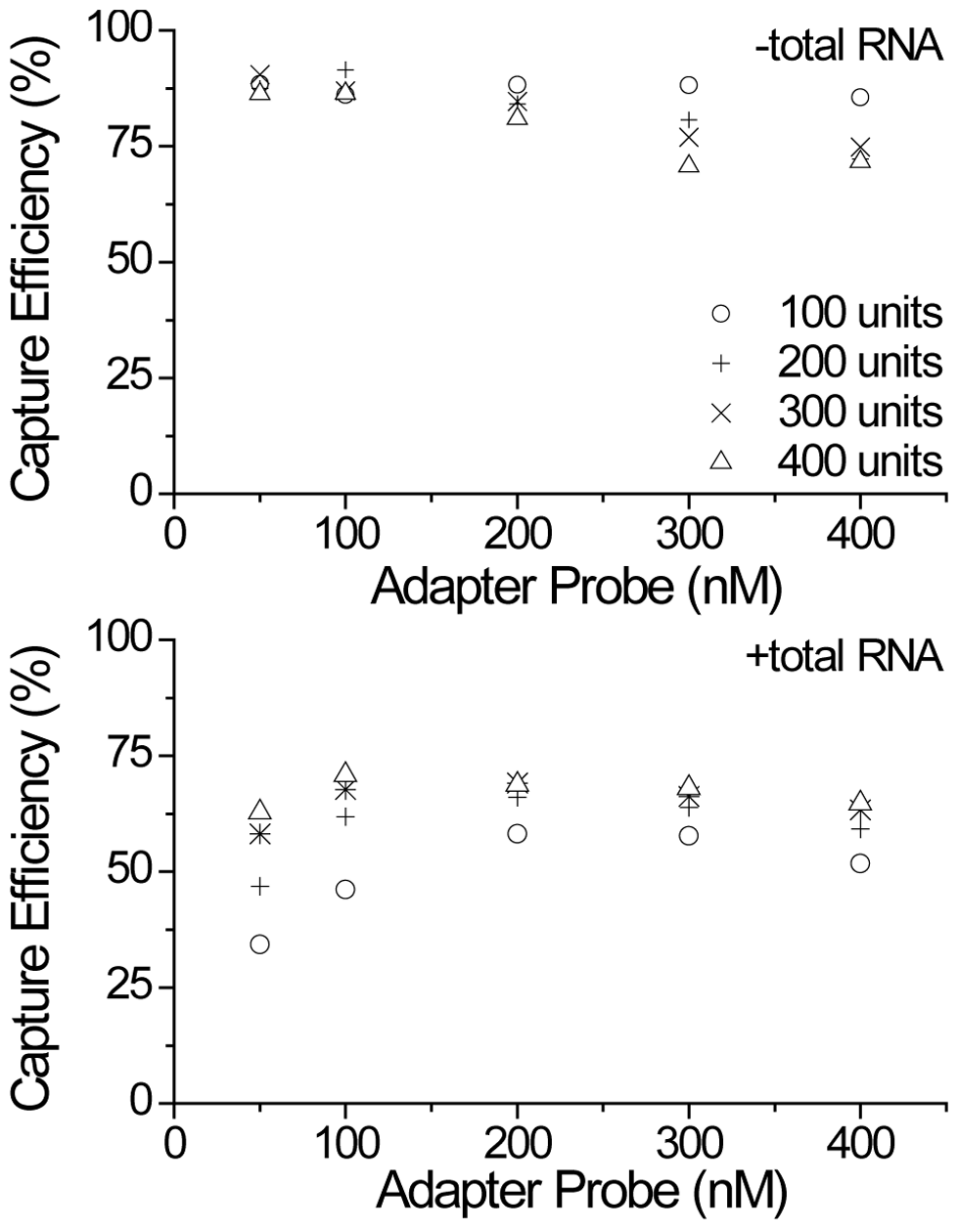
The adapter concentration and T4 Rnl2 TK amount were changed simultaneously to see their joint effect on the capture efficiency of miR-31. The experiment was performed under idealized buffer conditions and total RNA spiking conditions which lead to distinct conditions for optimum capture efficiency. Under spiking conditions, greater amounts of adapter and enzyme are necessary to obtain high capture efficiency.

Adapter concentration and ligase amount were optimized by quantifying their effects on the capture of miR-31, both in presence and absence of total RNA. **Figure 4** and **Figure S4 in File S1** show the effects of concurrently varying adapter concentration from 50 nM to 400 nM and ligase amount from 100 units to 400 units. In the absence of total RNA, little effect was seen by increasing either adapter or enzyme levels. Even at 50 nM adapter and 100 units of enzyme, ligation efficiency was 88% due to their high excess. However, under the same conditions in the presence of total RNA, ligation efficiency drops to 34% due to the large amounts of other RNA species in solution that the adapters can be ligated to and that ligase can spend time shaking hands with. Under these realistic spiking conditions, much larger amounts of adapter and enzyme were needed; 200–300 nM of adapter and 200 units of enzyme were needed to reach saturation. Although the highest capture efficiency was obtained with high amounts of both adapter and enzyme, high levels of at least one component also gave relatively high efficiencies.

### Incubation Time

**Figure 5:**
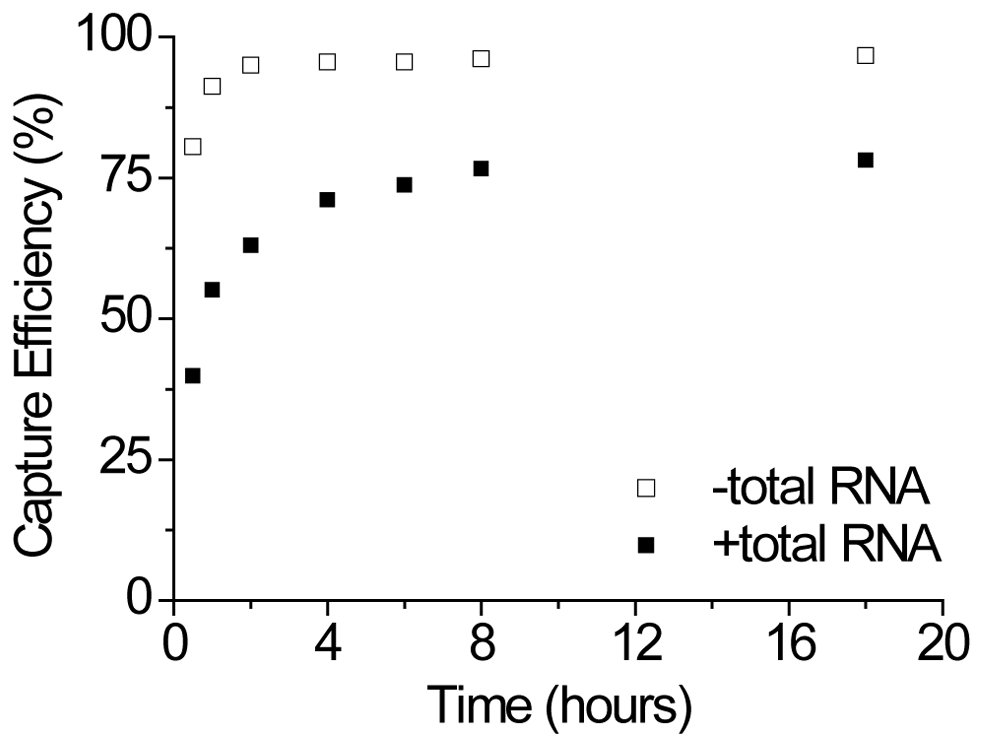
The ligation reaction was incubated at 25°C for 30 min to 18 hours to investigate the effect of time on the capture efficiency of miR-31 in idealized buffer conditions and total RNA spiking conditions. In idealized buffer conditions, the ligation reaches completion in <2 hours. Whereas in spiking conditions, the ligation does not reach full completion until >8 hours.

For the previous reactions, a 4 hour incubation was performed. We investigated whether this time was sufficient and whether it could be reduced. Using miR-31 as a model and the optimized protocol developed thus far, we tested incubation times from 30 minutes to 18 hours both in the presence and absence of total RNA. **Figure 5** and **Figure S5 in File S1** illustrate the microRNA capture efficiency as a function of time. As expected, the ligation reaction proceeded much faster in the absence of total RNA, reaching >80% in only 30 minutes and reaching plateau in <2 hours. When spiked into total RNA, plateau was not reached until >8 hours. This was likely due to the greater number of RNA species within the sample that the adapters could be ligated to. By 4 hours, ligation reached 92% of the plateau value, striking a good compromise between incubation time and ligation efficiency.

### Incubation Temperature

Secondary structure has been proposed as a main contributor to ligation bias [40], [41]. We investigated the effect of incubation temperature on ligation efficiency and ligation bias under the premise that elevated incubation temperatures could potentially reduce secondary structure interactions and alleviate bias. Initially, when we performed ligation experiments at 65°C using MthRnl, we did not see any significant difference when compared to ligation at 25°C using the T4 Rnl2 variants. However, in this case both the temperature and the ligase were changed. The intrinsic bias of the MthRnl could have swamped out any effects due to temperature.

**Figure 6:**
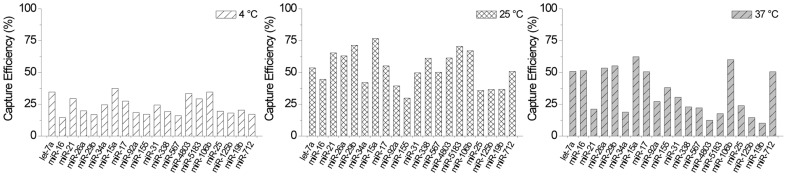
Comparison of incubation temperature on capture efficiency across the 20 microRNA panel. The ligation reaction was incubated for 18°C, 4 hours at 25°C or 4 hours at 37°C under total RNA spiking conditions.

We performed a second analysis using T4 Rnl2 TK while incubating at 4°C for 18 hours, 25°C for 4 hours, or 37°C for 4 hours. T4 Rnl2 TK is not thermostable and is denatured at 65°C so temperatures beyond 37°C were not tried. Incubation at 4°C allows for the reaction to proceed for an extended amount of time to compensate for decreased enzyme activity. Most enzymes recommend incubation at 25°C. We also performed ligation at 37°C to see if a modest increase in incubation temperature would have any effect on bias. **Figure 6** and **Figure S6 in File S1** show a graph and gel images, respectively, of the ligation efficiency for all 20 microRNA in our test panel using the 4°C, 25°C, and 37°C conditions when spiked into 500 ng of total RNA.

The highest efficiency and lowest bias were seen at the 25°C condition where ligation efficiency across the 20 microRNA panel was 53.1% AVG ±13.7% SD. When the incubation temperature was decreased to 4°C, the ligation efficiency across the panel decreased to 23.6% AVG ±7.2% SD. Despite the increased ligation time (18 hours vs. 4 hours), the decreased enzyme activity at 4°C led to significantly reduced ligation efficiency. Interestingly, the capture efficiency CV for the 4°C condition (31% CV) was only slightly worse than at 25°C (26% CV), indicating that temperature does not have a large impact on bias across this range. The pattern in capture efficiency for each individual microRNA in the panel was fairly similar for the two conditions except that the capture efficiency at 25°C was 2× higher in most cases. When the incubation temperature was increased to 37°C, the ligation efficiency across the panel dropped to 34.7% AVG ±17.7% SD. The higher incubation temperature reduced the ligase activity but unexpectedly increased the bias over both the 4°C and 25°C conditions to nearly 51% CV. Incubation temperature likely results in a combination of effects on ligase activity, ligase degradation, and secondary structure formation that impact ligation efficiency in a complex manor. With a few exceptions, the pattern in ligation efficiency across the panel was generally similar to the 4°C and 25°C conditions.

### Adapter Design

**Figure 7:**
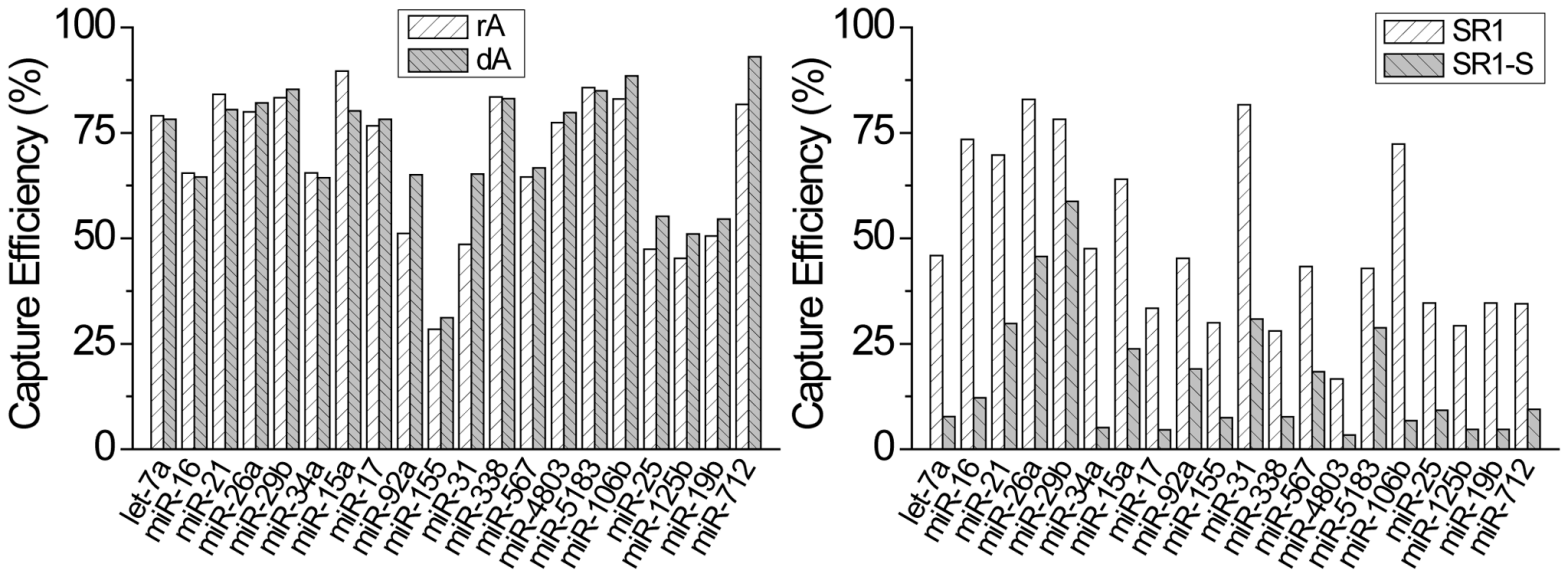
Comparison of capture efficiency across the 20 microRNA panel spiked into 500 ng of total RNA using 4 different adapter designs. The rA and dA adapters have identical sequences based on modified modban design except the 5′ base is either RNA or DNA as indicated. The SR1 and SR1-S adapters are taken from Zhuang *et al* (39). T4 Rnl2 TK shows no preference for DNA or RNA at the ligation site. However, overall capture efficiency and bias were significantly worse for the SR1 and SR1-S adapters.

Thus far, we've investigated the use of reaction conditions to suppress the intrinsic bias of T4 Rnl2 TK. Adapter design can also play a large part in ligation bias due to primary sequence and/or secondary structure effects. However, the differences in microRNA capture efficiency seen across published studies illustrate the unpredictable nature of these interactions [36], [37], [40], [41]. For example, even when adapters were logically designed to either eliminate inhibitory secondary structures or promote favorable interactions, only modest improvements, if any, were seen [41]. In **Figure 7** and **Figure S7 in File S1**, we performed adapter ligation on the 20 microRNA panel in spiking conditions (500 ng total RNA) using four different adapter sequences. First, we synthesized two versions of our modified modban adapter [44] to test whether having RNA or DNA as the 5′ residue would affect ligation efficiency or bias. As the ligases used herein are RNA ligases used for single stranded blunt end ligations, it is possible that the ligases will have a preference for ligating RNA versus DNA. The rA version contains a ribo-A as the 5′ base while the dA version contains a dexoyribo-A as the 5′ base. As seen in Figure 7 left, both of our modified modban adapters achieved high ligation efficiencies with low bias. No significant difference was seen between the rA or dA versions of the adapter, indicating that T4 Rnl2 TK displays no real preference for RNA-RNA ligation or RNA-DNA ligation. The rA adapter had a capture efficiency of 68.5%±17.6% (AVG ± SD) across the 20 microRNA panel while the dA adapter had a capture efficiency of 71.6%±15.4% (AVG ± SD). Even under spiking conditions, the capture efficiencies surpass what previous publications were able to achieve using complex adapter pool strategies in idealized buffer conditions. Using the dA adapter, 19 microRNA were captured at >50% with the lowest still being captured at 31%. We attribute the low capture efficiency of miR-155 to degradation or synthesis problems. We re-synthesized miR-155 multiple times. Immediately after receiving the target, high ligation efficiencies were obtained which would slowly degrade over time. MiR-155 was the only target we saw this effect with.

Encouraged by the previous results, we synthesized the SR1 and SR1-S adapters used by Zhuang [41] and Hafner [40] to test whether our reaction would work equally well with other adapter designs. SR1 is a standard adapter commonly used in Illumina's sequencing products. SR1-S was designed by Zhuang to reduce ligation bias by eliminating inhibitory secondary structure interactions. As seen in Figure 7 right, the overall ligation efficiency and bias were much poorer with both of these adapter designs. The SR1 capture efficiency across the 20 microRNA panel was 49.4%±20.6% with only 7 microRNA captured at >50%. The SR1-S adapter fared even worse with only 1 microRNA being captured at >50%. SR1-S had a capture efficiency of 16.9%±15.2% (AVG ± SD) across the panel. This result parallels that reported by Zhuang where the SR1-S adapter unexpectedly performed much worse than the SR1 adapter and failed to improve ligation efficiencies despite eliminating secondary structure interactions. Given the current reaction conditions and microRNA test panel, our modified modban adapter appears to work significantly better than the SR1 adapter. It is possible that the ligation reaction conditions can be optimized specifically for the SR1 adapter but we did not attempt this.

### High Efficiency and Low Bias microRNA Capture

**Figure 8:**
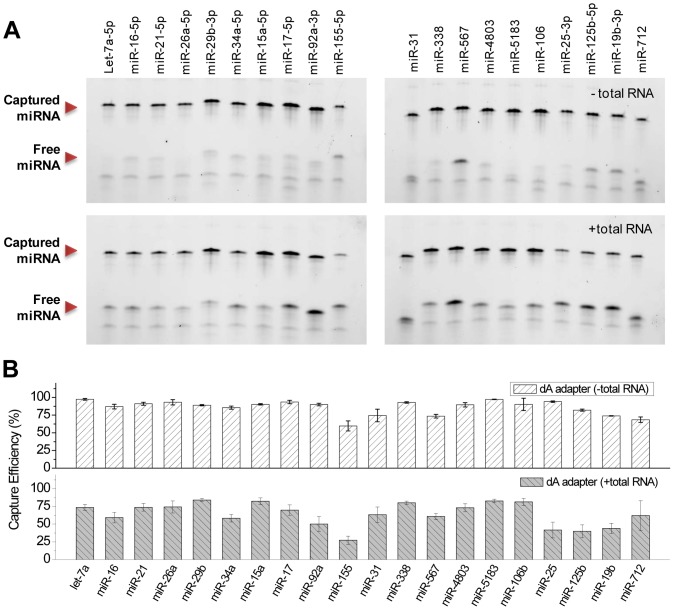
The optimized capture efficiency for the 20 microRNA panel in idealized buffer conditions and total RNA spiking conditions. The data is presented as representative gel images and as graphs based on image analysis of 3 sets of independent experiments. High capture efficiency and low bias are obtained across the panel both in idealized buffer conditions and total RNA spiking conditions.

Based on the optimized conditions determined in previous experiments, we performed ligation on the 20 microRNA panel using 300 units of T4 Rnl 2 TK, 25% PEG, 200 nM adapter incubated for 4 h at 25°C. Ligation was performed both in idealized buffer and in total RNA spiking conditions as shown in **Figure 8**. Each experiment was performed in triplicate. In the absence of total RNA, the capture efficiency across the 20 microRNA panel was 86% AVG ±10% SD. No microRNA was captured at less than 60% with 18 of 20 microRNA being captured at 70% or greater. This is a considerable improvement over the initial conditions of 20%±25% (AVG ± SD). Optimization of various parameters such as PEG, adapter, enzyme levels, and incubation time led to an increase in overall ligation efficiency and the concurrent benefit of effectively reducing ligation bias. Saturating levels of each parameter tend to push all of the competing reactions toward completion, regardless of moderate differences in thermodynamic equilibrium and kinetics. Thus, as the capture efficiency of all the microRNA increases and approaches 100%, bias is concurrently reduced since capture saturates at 100%.

When compared to previous studies performed in idealized buffer background, a significant improvement in average capture efficiency and bias was seen. For example Hafner *et al* reported that miR-16 (92%), miR-21(66%), miR-155 (90%), and miR-567 (73%) were well captured while miR-31 (48%) and miR-338 (3%) were less well captured [40]. In our process, all of these microRNA are uniformly captured at 87%, 91%, 60%, 74%, 74%, and 93%, respectively. Similar results are seen when compared to Zhuang *et al* [41]. They reported let-7a (100%), miR-31 (87%), and miR-567 (56%) to be well captured while miR-4803 (1%), miR-5183 (42%), and miR-712 (8%) were poorly captured. All of these microRNA species are well captured with our methods at 97%, 74%, 74%, 90%, 97% and 69%, respectively. In addition, miR-106, which could not be captured consistently by Jayaprakash *et al* under any conditions, is now captured at 90% [36].

These differences are further pronounced when comparing microRNA capture in realistic spiking conditions. The presence of total RNA likely reduces capture efficiency due to the aforementioned competitive reactions. Under realistic spiking conditions in presence of total RNA, our capture efficiency is slightly decreased and bias is slightly increased with a capture efficiency of 64% AVG ±17% SD across the panel. 16 of 20 microRNA have capture efficiencies greater than 50%. Only miR-155 had a low capture efficiency (27%). We experienced repeatability problems with miR-155, likely due to synthesis and degradation, as previously described. In comparison, Zhuang *et el* reported significantly reduced capture efficiencies under spiking conditions with efficiencies of 19%, 17%, 9%, 2% and 4% for let-7a, miR-31, miR-567, miR-4803, and miR-5183, respectively [41]. To resolve this, they proposed a random adapter pool approach to average out the low capture efficiencies. However, the capture efficiencies for the pooled approach still remained relatively low at 83%, 27%, 8%, 9%, and 14%, respectively. A recent study by Zhang *et al* [43] has also used randomized adapter pools to reduce bias. Bias was reduced to1.8-fold from the expected frequency across a 29 microRNA panel. In comparison, the current method uses only a single adapter and achieves highly efficient and low bias capture levels of 74%, 63%, 61%, 73%, and 83% for let-7a, miR-31, miR-567, miR-4803, and miR-5183. In fact, our spiking capture efficiencies surpass what has been demonstrated by previous studies under idealized buffer conditions.

The experiments shown in Figure 8 were performed using a dA adapter labeled with Cy5. Additionally, we also performed this experiment in triplicate using an rA adapter labeled with Cy5 and an rA adapter blocked with ddC (data not shown). In each case, similar results were obtained, demonstrating that the adapter ligation process is highly repeatable and robust. This is further evidenced by the low average experiment-to-experiment SD for each microRNA seen in Figure 8, which was 3% in idealized buffer and 7% in total RNA spiking. miRNA capture efficiency is also consistent across a broad range of miRNA input levels as seen in **Figure S8 in File S1**.

## Conclusion

Based on results across the 20 microRNA panel, we believe that ligation bias can be largely suppressed through suitable ligation optimization methodology, as opposed to solely focusing on adapter design. It is critical to design the reaction methodology to optimize for bias rather than just ligation efficiency. It is also critical to optimize using a panel of microRNA targets, rather than a single species, and to perform experiments both in idealized buffer and in realistic spiking conditions. Total RNA spiking has a large impact on the optimal reaction conditions, likely due to a number of competitive and inhibitive effects. Using this methodology, we were able to capture a panel of 20 microRNA at 86% AVG ±10% SD (12% CV) in buffer and 64%±16.5% SD (26% CV) in spiking conditions. This variability is below the typical variability in PCR amplification efficiency and means that expression profiles will likely vary very little due to this step. In contrast to previous methods that incorporated randomized adapter pools, the current method achieves low bias using only a single adapter sequence to capture all 20 microRNA. The elimination of adapter pools can greatly simplify downstream assay design. Key to this process was the use of very high PEG levels (25%) to suppress bias, particularly under total RNA spiking conditions. This PEG level is about double that which is typically recommended (10-15%). Molecular crowding by PEG has been demonstrated to affect both RNA enzyme activity [53] and RNA folding [54], [55]. As discussed, previous studies have that indicated secondary structure interactions as a primary contributor to ligation bias. Thus, it is likely that the stabilization of RNA folding by high PEG can play a significant role in suppressing bias.

Through this process we have also discovered that variability in the adapter and microRNA synthesis can lead to significant artifacts. The oligonucleotides that we ordered were always HPLC purified and had specified yields of >80%, yet we saw large variations in capture efficiency across different batches of adapters and microRNA targets. 3 microRNA targets that initially appeared poorly captured became consistently well captured after the targets were re-synthesized. However, miR-155 and, to a lesser degree miR-712, behaved increasingly erratically as the targets aged and required multiple re-synthesis rounds to obtain optimal results. Interestingly, all the other microRNA targets remained very stable over time. It is possible that some of the variability and inconsistencies seen in previous papers could be also attributed to synthesis issues. While the 20 microRNA panel used herein is not exhaustive of all microRNA species, we feel it is a good representative snapshot of what can be expected.

## Supporting Information

File S1 **Table S1, Figures S1-S8.**
(DOCX)
