# Supplementary material for: Elimination of Ligation Dependent Artifacts in T4 RNA Ligase to Achieve High Efficiency and Low Bias MicroRNA Capture
Source: PLoS One. 2014 Apr 10;9(4):e94619. doi: 10.1371/journal.pone.0094619 (PMC3983213; doi:10.1371/journal.pone.0094619)
Supplement: File S1 — Table S1, Figures S1-S8. (DOCX) [file pone.0094619.s001.docx]

Elimination of Ligation Dependent Artifacts in T4 RNA Ligase to Achieve High Efficiency and Low Bias microRNA Capture

Yunke Song^1^, Kelvin J Liu^2,3,*^ and Tza-Huei Wang^1,2,*^

^1^ Biomedical Engineering Department, Johns Hopkins University, Baltimore, MD, 21218, USA

^2^ Mechanical Engineering Department, Johns Hopkins University, Baltimore, MD, 21218, USA

^3^ Circulomics Inc, Baltimore, MD, 21211, USA

* To whom correspondence should be addressed: Tel: +1 410 516 7086; Fax: +1 410 516-7254; Email: thwang@jhu.edu Correspondence may also be addressed to: Tel: + 1 626 202 4825; Email: kliu@circulomics.com.

# Supplementary Data

| **Name** | **Sequence 5'-> 3'** |
| --- | --- |
| *let-7a* | 5'- /5Cy3/rUrG rArGrG rUrArG rUrArG rGrUrU rGrUrA rUrArG rUrU -3' |
| *miR-106b* | 5'- /5Cy3/rUrA rArArG rUrGrC rUrGrA rCrArG rUrGrC rArGrA rU -3' |
| *miR-125b* | 5'- /5Cy3/rUrC rCrCrU rGrArG rArCrC rCrUrA rArCrU rUrGrU rGrA -3' |
| *miR-155* | 5'- /5Cy3/rUrU rArArU rGrCrU rArArU rCrGrU rGrArU rArGrG rGrGrU -3' |
| *miR-15a* | 5'- /5Cy3/rUrA rGrCrA rGrCrA rCrArU rArArU rGrGrU rUrUrG rUrG -3' |
| *miR-16* | 5'- /5Cy3/rUrA rGrCrA rGrCrA rCrGrU rArArA rUrArU rUrGrG rCrG -3' |
| *miR-17* | 5'- /5Cy3/rCrA rArArG rUrGrC rUrUrA rCrArG rUrGrC rArGrG rUrArG -3' |
| *miR-19b* | 5'- /5Cy3/rUrG rUrGrC rArArA rUrCrC rArUrG rCrArA rArArC rUrGrA -3' |
| *miR-21* | 5'- /5Cy3/rUrA rGrCrU rUrArU rCrArG rArCrU rGrArU rGrUrU rGrA -3' |
| *miR-25* | 5'- /5Cy3/rCrA rUrUrG rCrArC rUrUrG rUrCrU rCrGrG rUrCrU rGrA -3' |
| *miR-26a* | 5'- /5Cy3/rUrU rCrArA rGrUrA rArUrC rCrArG rGrArU rArGrG rCrU -3' |
| *miR-29b* | 5'- /5Cy3/rUrA rGrCrA rCrCrA rUrUrU rGrArA rArUrC rArGrU rGrUrU -3' |
| *miR-31* | 5'- /5Cy3/rArG rGrCrA rArGrA rUrGrC rUrGrG rCrArU rArGrC rU -3' |
| *miR-338* | 5'- /5Cy3/rUrC rCrArG rCrArU rCrArG rUrGrA rUrUrU rUrGrU rUrG -3' |
| *miR-34a* | 5'- /5Cy3/rUrG rGrCrA rGrUrG rUrCrU rUrArG rCrUrG rGrUrU rGrU -3' |
| *miR-4803* | 5'- /5Cy3/rUrA rArCrA rUrArA rUrArG rUrGrU rGrGrA rUrUrG rA -3' |
| *miR-5183* | 5'- /5Cy3/rUrA rUrUrU rGrGrA rCrArA rArUrU rUrGrA rGrUrC rA -3' |
| *miR-567* | 5'- /5Cy3/rArG rUrArU rGrUrU rCrUrU rCrCrA rGrGrA rCrArG rArArC -3' |
| *miR-712* | 5'- /5Cy3/rCrU rCrCrU rUrCrA rCrCrC rGrGrG rCrGrG rUrArC rC -3' |
| *miR-92a* | 5'- /5Cy3/rUrA rUrUrG rCrArC rUrUrG rUrCrC rCrGrG rCrCrU rGrU -3' |
|  |  |
| Modban Adapter* | 5'- CTGTAGGCACCATCAAT -3' |
| SR1 Adapter | 5'- TCGTATGCCGTCTTCTGCTTG-NH_2_ -3' |
| SR1-S Adapter | 5'- TCGTATGCCGTCACGGCTCGA-NH_2_ -3' |

**Table S1.** Adapter probe and microRNA sequences. The adapter probe was based on a modified version of the modban adapter.

**Figure S1.** The 19 nt Cy5-labelled adapter probe was serially diluted and analyzed by 15% denaturing PAGE. A Cy5 scan was performed on a Typhoon imager, and image analysis was used to determine band intensity. The experiment was repeated 3 times. The fluorescence was linear from <5e-18 mol to >1e-11 mol. The weighted least squares fit shown in red has an R^2^ ~0.999.

**Figure S2**. Image analysis was performed on the gel data in Figure 2 to obtain capture efficiencies for each of the 20 microRNA in the panel using T4 Rnl2 T, T4 Rnl2 TK, T4 Rnl2 TKQ, and Mth Rnl. The reactions were performed using the vendor recommended protocols in each case.

**

**Figure S3.** Raw PAGE images depicting the effects of PEG on capture efficiency for *miR-31*, *miR-155* and *miR-4803* in idealized buffer conditions (-RNA) and spiked into 500 ng of total RNA (+RNA). PEG was varied from 0% to 35%. Image analysis was performed on this data to obtain Figure 3.

**Figure S4.** Raw PAGE images depicting the effects of adapter probe concentration and enzyme amount on the capture efficiency of *miR-31* in idealized buffer conditions and spiked into 500 ng of total RNA. Image analysis was performed on this data to obtain Figure 4.

**Figure S5.** Raw PAGE images depicting the effects of incubation time on the capture efficiency of *miR-31* in idealized buffer conditions and spiked into 500 ng of total RNA. Image analysis was performed on this data to obtain Figure 5.

**Figure S6.** Raw PAGE images depicting the effects of incubation temperature on the capture efficiency and bias across the 20 microRNA panel. Ligation was performed at 4 °C, 25 ºC, and 37 °C under identical conditions spiked into 500 ng of total RNA. Image analysis was performed on this data to obtain Figure 6.

**Figure S7.** Raw PAGE images depicting the effects of 4 different adapter probe designs on the capture efficiency and bias across the 20 microRNA panel under spiking conditions. The rA and dA adapter probes are based off of a modified modban probe design with RNA-A vs. DNA-A as the first letter, respectively. The SR1 and SR1-S probes are taken from Zhuang *et al* (39) and Hafner *et al* (38). Image analysis was performed on this data to obtain Figure 7.


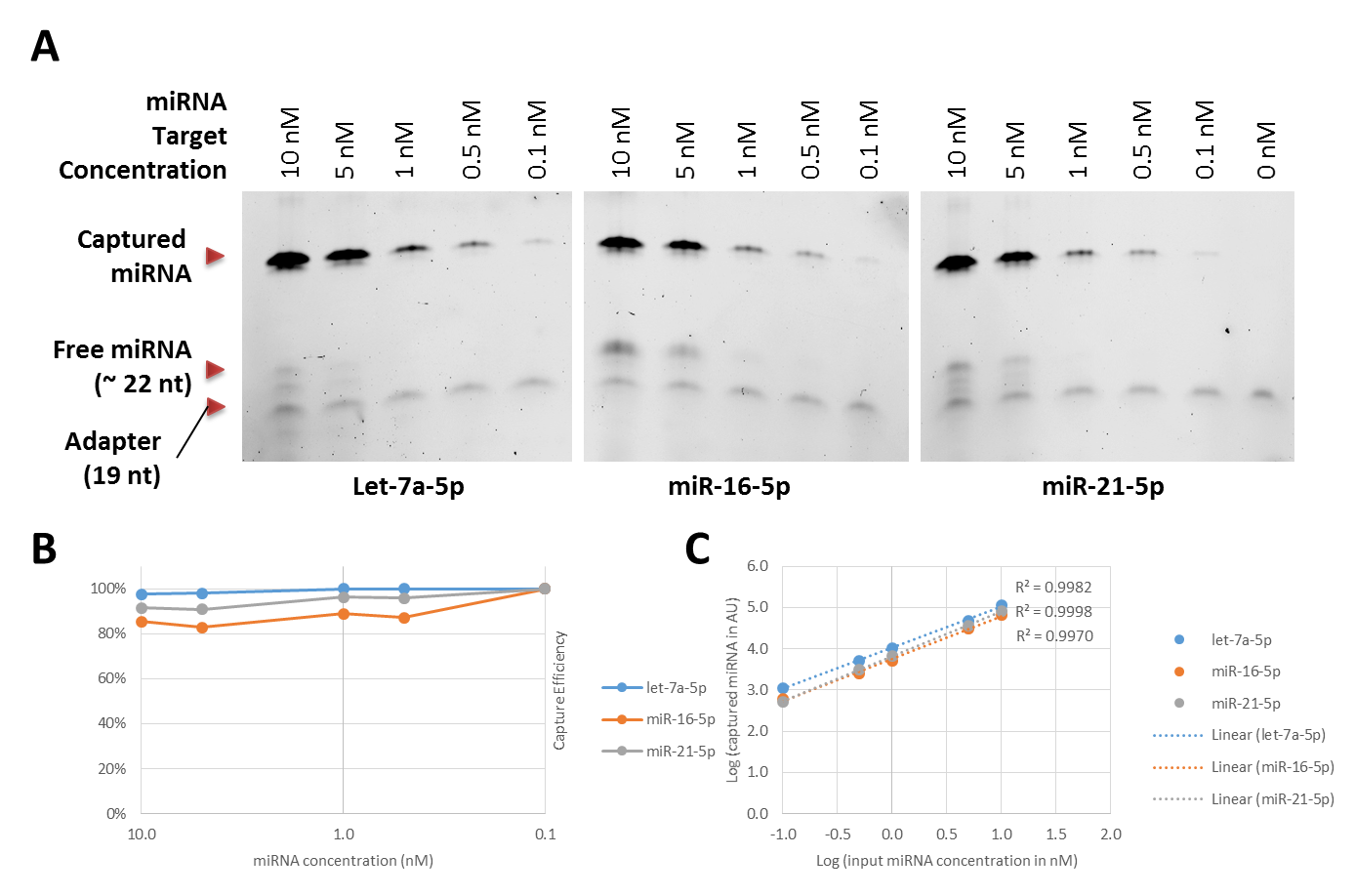


**Figure S8.** (A) *Let-7a*, *miR-16*, and *miR-21* were serially diluted from 10 nM - 0.1 nM and captured using the optimized method. The reaction products were then analyzed by denaturing PAGE. (B) The capture efficiency was calculated and plotted against the input miRNA (capture efficiency = captured miRNA / (captured miRNA + free miRNA)). (C) The amount of captured miRNA was plotted against the input miRNA level. Both data indicate that the capture efficiency stays constant across a wide range of input miRNA levels.
